# Contrast-Enhanced MRI Texture Parameters as Potential Prognostic Factors for Primary Central Nervous System Lymphoma Patients Receiving High-Dose Methotrexate-Based Chemotherapy

**DOI:** 10.1155/2019/5481491

**Published:** 2019-11-12

**Authors:** Chaoyue Chen, Hongyu Zhuo, Xiawei Wei, Xuelei Ma

**Affiliations:** ^1^State Key Laboratory of Biotherapy and Cancer Center, West China Hospital, Sichuan University and Collaborative Innovation Center for Biotherapy, Chengdu, China; ^2^Department of Neurosurgery, West China Hospital, Sichuan University, Chengdu, China; ^3^Department of Biotherapy, Cancer Center, West China Hospital, Sichuan University, Chengdu, China

## Abstract

**Introduction:**

The purpose of this study was to evaluate the prognostic value of texture features on contrast-enhanced magnetic resonance imaging (MRI) for patients with primary central nervous system lymphoma (PCNSL).

**Methods:**

In this retrospective study, fifty-two patients diagnosed with PCNSL were enrolled from October 2010 to March 2017. The texture feature of tumor tissue on the histogram-based matrix (histo-) and the grey-level co-occurrence matrix (GLCM) was retrieved by contrast-enhanced T1-weighted imaging before any antitumor treatment. Receiver operating characteristic curve analyses were performed to obtain their optimal cutoff values, based on which we dichotomized patients into subgroups. The Kaplan–Meier analyses were conducted to compare overall survival (OS) of subgroups, and multivariate Cox regression analyses were used to determine if they could be taken as independent prognostic factors.

**Results:**

Ten texture features were extracted from the MR image, including Energy, Entropy, Kurtosis, Skewness on the histogram-based matrix, and Correlation, Contrast, Dissimilarity, Energy, Entropy, and Homogeneity on the grey-level co-occurrence matrix. Three of them (GLCM-Contrast, GLCM-Dissimilarity, and GLCM-Homogeneity) are shown to be significant in relation to overall survival (OS). The multivariate Cox regression analyses suggest that GLCM-Homogeneity could be taken as independent predictors.

**Conclusions:**

The texture features of contrast-enhanced magnetic resonance imaging (MRI) could potentially serve as prognostic biomarkers for PCNSL patients.

## 1. Background

Primary central nervous system lymphoma (PCNSL) is an uncommon type of tumor, accounting for about 1% non-Hodgkin's lymphomas (NHLs) and 3% primary cerebral tumors. Characterized as a type of aggressive brain tumor, the lymphoma tissues confine to the central nervous system (CNS), including the brain, spinal cord, leptomeninges, and eyes [1, 2]. The diffuse large B-cell lymphoma (DLBCL) constitutes nearly 95% of PCNSL while <4% of PCNSL are of T-cell origin [3, 4]. Chemotherapy is the basic treatment for PCNSL, and high-dose methotrexate- (HD-MTX-) based chemotherapy is highly recommended due to the ability in significantly increasing the patient's overall survival time according to recent studies [5, 6]. With the advance in treatment over the last 20 years, the five-year survival rate for patients treated with HD-MTX is approximately 15%∼20%, especially for those at young age or with tumor tissue with good performance status.

Texture analysis (TA) is a statistical method of analyzing the region of interest (ROI) of an image from plain radiographs, magnetic resonance imaging (MRI), and computed tomography (CT) [7]. Based on the mathematical calculation on spatial organization and distribution of grey values, the TA could provide the nonvisual information to assist in diagnosis and to predict the prognosis. Recent studies illustrated that the MRI texture features could be potential prognostic biomarkers for patients with various types of cancer, including breast cancer, rectal cancer, and soft-tissue sarcomas [8–10]. Inspired from these studies, we performed this research to investigate the relationship between MRI texture features and the survival of patients with PCNSL, and to determine if MRI texture features could be reliable parameters in predicting patient survival.

## 2. Method

### 2.1. Patient Selection

The patients involved in this study were selected from the neurosurgery department of our institution, who were diagnosed and treated between October 2010 and March 2014. Initially, we reviewed all the electronic medical records to evaluate if the potential patients were potentially qualified for this study, and to collect clinical information which could have an impact on patient survival. DLBCL is the most common lymphoma of pathological subtype, bringing the dilemma of scarce information on patients with other lymphoma subtypes and unconvincing statistical analysis on pathological subtype. So we decided to include only DLBCL patients in this study while patients with other types of lymphoma were excluded. The inclusive criteria were as follows: patients (a) with pathological confirmation; (b) with elaborate medical record on the progress of diagnosis and treatment; (c) with an available MRI imagine before antitumor treatment. The exclusive criteria included patients: (a) with a history of surgical intervention of intracranial disease; (b) clinical proof on other diseases that could decrease the survival rate significantly (such as infection, coronary disease, and uremia). Based on this strategy, we made a preliminary list and collected follow-up information from the database. Other clinical parameters, such as age, gender, chemotherapy regime, and radiotherapy regime, were also recorded.

It is worth noting that the role of surgery was controversial in the recent years. The dominant perception was that surgery was not recommended except for biopsy because of the mutual view among the neurosurgeons that there is no benefit from resection for patients. In our study, the neurosurgeons agreed with the standpoint and the purpose of resection for patients was only for biopsy. However, some patients undertook subtotal resections (STRs) due to the radiological misdiagnosis of nontypical lymphoma, while pathologically confirmed by intraoperative frozen section diagnosis during operation.

The endpoint of this study was overall survival (OS), which was calculated as the time from the date of histological diagnosis to the date of tumor-related death or the last time access. Based on the results of telephone follow-ups or medical record, we documented their survival condition until Mar 2019. This study was approved by the medical ethics committee of the institution.

### 2.2. MRI Acquisition

The MRI scan was conducted via a 3 Tesla GE MRI system with an 8-channel phase-array head coil. The subjects were asked to keep their eyes closed, stay relaxed, and reduce movement. The parameters of the contrast-enhanced T1-weighted imaging were: time repetition = 2000 ms, time echo = 30 ms, flip angle = 90°, slice thickness = 5 mm (no slice gap), field of view = 240 × 240 mm^2^, 30 axial slices, and 200 volumes in each run.

### 2.3. MRI Image Texture Feature Analysis

**Figure 1:**
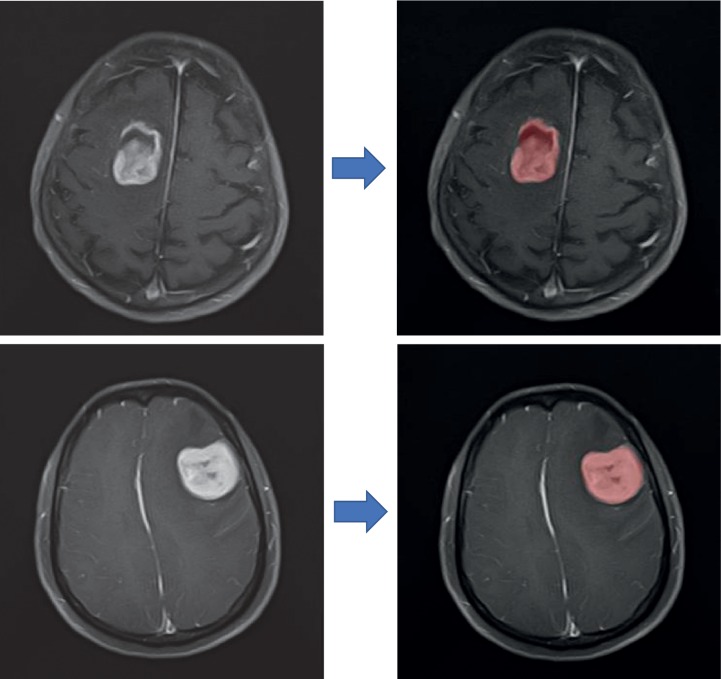
The screenshot of the texture features extraction.

The MRI texture features were extracted by two certified neurosurgeons using LIFEx software (http://www.lifexsoft.org.) with assistance of certificated nuclear medicine physicians (Figure 1). It is worth noting that all MR images used for texture analysis were performed before any antitumor treatment bringing down the influence of radio or chemical therapy on texture analysis. As per the instruction of software, 3D ROI was extracted by manually drawing along the lesion slice by slice [11]. Based on feedback from surgeons and statistics of texture features, we retrieved TA on axial T1-weighted imaging after contrast was chosen to perform survival analysis due to rather good description on the lesion location. The Mann–Whitney *U* test suggested that there was no statistical difference in features extracted by two users (Supplement [Supplementary-material supplementary-material-1]). Eventually, textures of imaging were extracted into a dataset for further analysis from two matrixes, including Energy, Entropy, Kurtosis, Skewness on the histogram-based matrix, and Correlation, Contrast, Dissimilarity, Energy, Entropy, Homogeneity on the grey-level co-occurrence matrix. Partial volume effect correction was not performed.

### 2.4. Statistical Analysis

In this study, we performed our analyses using MedCalc statistics and SPSS statistics version 21 (IBM Corp., Chicago). The optimal cutoff value was determined at the point of the maximal Youden's index with receiver operating characteristic (ROC) analyses [12]. Kaplan–Meier Curve was performed to illustrate the relationship between specific features and patient survival. Moreover, multivariate analyses were conducted with the Cox proportional hazards regression model to determine their value of being independent predictors. The correlations between features were indicated by Pearson Correlation analysis.

## 3. Results

### 3.1. Patient Selection

A total number of 52 patients were selected from the database to be involved in this study. The mean age of patients was 53.57 years (range, 29∼74 years), and the sex ratio was 29 : 23 (29 males and 23 females). Two different chemotherapy regimens were applied to these patients based on their situation, high-dose MTX-based chemotherapy (HD-MTX+), and R-CHOP+MTX chemotherapy regimen. The whole brain radiotherapy (WBRT) was applied to 11 patients as radiotherapy. The detailed characteristics of patients are summarized in Table 1. One-way ANOVA analysis suggested that the radiotherapy was significantly correlated to patient survival while other clinical parameters were not (*p*=0.032).

### 3.2. MRI Texture Features and Survival Analysis

**Figure 2:**
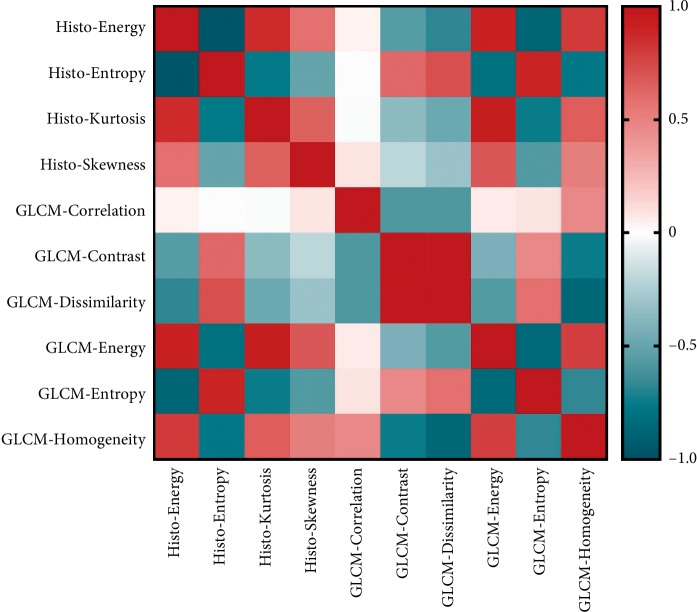
HOTMAP of MRI textural parameters.

Considering the complicated relationship between texture features, Pearson correlation was conducted first to evaluate the association between features. The result suggested most of the MRI textures were correlated with each other significantly (Figure 2).

**Figure 3:**
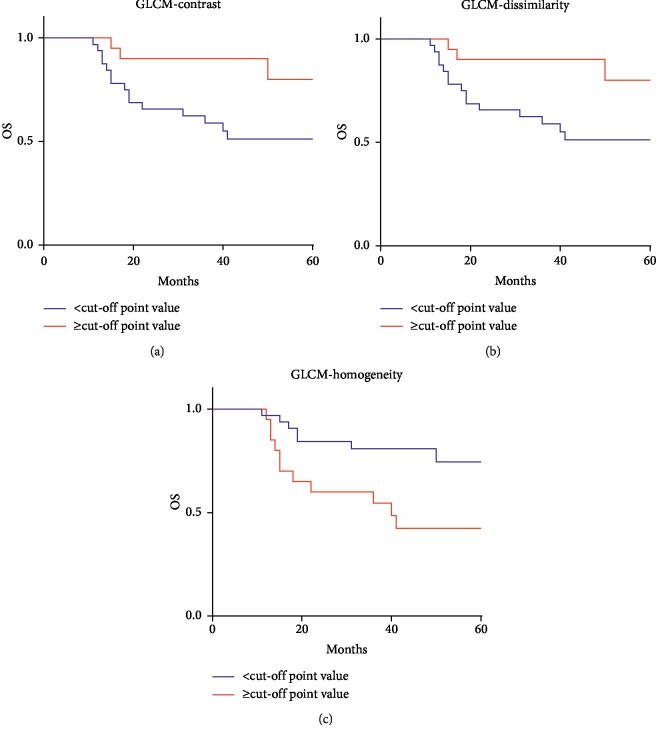
Kaplan–Meier survival curves on the texture parameters of (a) GLCM-Contrast, (b) GLCM-Dissimilarity, and (c) GLCM-Homogeneity. Patients with (a) GLCM-Contrast >121.1726, (b) GLCM-Dissimilarity >7.8085, and (c) GLCM-Homogeneity <0.2864 show better survival, with log-rank tests showing *p* values of 0.027, 0.027, and 0.014, respectively.

The results of ROC analyses showed that 3 of MRI texture features (GLCM-Contrast (*p*=0.016), GLCM-Dissimilarity (*p*=0.015), and GLCM-Homogeneity (*p*=0.039)) were found to be significantly related to OS (Table 2). Then we conducted K-M analyses to compare their value to predict OS of patients. The K-M survival analyses demonstrated that the survival rate would be worse in the patients with lower GLCM-Contrast (*p*=0.027), lower GLCM-Dissimilarity (*p*=0.027), and higher GLCM-Homogeneity (*p*=0.014) than the optimal cutoff values (Figure 3). Subsequently, multivariate Cox proportional hazards regression analyses were performed to see if they could be considered as independent prognostic parameters. The age of patients and radiotherapy regime were also included into the model considering the impact of age on patient survival. The results showed that GLCM-Homogeneity (*p*=0.021, HR = 3.075) had potential to be an independent factor while radiotherapy (*p*=0.559), GLCM-Contrast (*p*=0.242), and GLCM-Dissimilarity (*p*=0.242) do not (Table 3).

## 4. Discussion

Our study investigated the relationship between texture features and prognosis of PCNSL patients, suggesting that parameters on contrast-enhanced MRI could potentially serve as parameters to predict the prognosis of patients diagnosed with DLBCL, and GLCM-Homogeneity have potential to be a clinically independent predictor.

The prognostic prediction of PCNSL is essential for physicians as they need the information to adapt therapeutic schedule and guide expectations for patients based on their prognosis. Nowadays, the clinicians make prediction on patient survival based on several parameters, including age, Karnofsky performance status (KPS), location of lesions, and blood test results. However, the prognostic value of radiological parameters is still unclear and needed research to verify. In our research, we demonstrated the texture analysis had potential to predict prognosis of PCNSL, technically for patients with DLBCL, hoping to introduce a new set of parameters to assist physicians in making more accurate and precious decisions. To the best of our knowledge, our research is the first one demonstrating the statistically significant association between radiomic on contrast-enhanced MRI and prognosis of PCNSL patients.

Texture analysis was considered as a novel method with great potential and has been developed rapidly in recent years. Given that the MRI is a routine examination for intracranial tumors, TA would provide an efficient method to predict patient survival with advantages of convenience and no additional cost. The value of TA in diagnosis and prognosis of intracranial tumors has been indicated before. One study explored the ability of TA in distinguishing SFT/HPC from meningioma, suggesting that TA could assist surgeons in designing therapeutic and surgical strategy [13]. Another study investigated the diagnostic value of MRI texture features in the discrimination of glioblastoma (GBM) and PCNSL, two of which shared similar visual characteristics on MRI [14, 15]. The prognostic value of MRI texture has also been illustrated but with relatively limited number. Only one study illustrated the relationship between texture features on MR-perfusion image and survival of PCNSL patients, but negatively association was observed [16]. In our study, the relationship between texture parameters and survival were conducted on contrast-enhanced T1-WI, and the results showed that GLCM-Homogeneity could be considered as an independent prognostic predictor.

The mechanism for TA as prognostic predictors is that texture features are correlated with tumor biology, and the variation on features indicated the tumor progression in this specific malignant lesion. The appearance of PCNSL on MRI is characteristically described as hypercellular and represents homogenous enhancement with or without necrosis after administration of contrast [17]. Theoretically, the characteristics of tumors could be depicted by TA mathematically as spatial frequency, statistical parameters, or structural primitives [18]. The possible explanation for the GLCM-Homogeneity as independent predictors was conceptually from the meaning of the feature that it describes the homogeneity of grey-level voxel pairs of images. The variations on parameters might reflect the pathological procedure of tumors such as neovascularization and hypoxia, which eventually result in necrosis represented as in MRI and usually are taken as the sign of poor prognosis.

TA could also have potential to serve as a novel imaging technology in the biology research with the ability in analyzing nonvisual information. The ability of TA on conventional MRI image in discriminating the pathological subtype of intracranial disease has been reported in a previous study [19]. Some researchers have also reported that the profiling of the histogram-based matrix on diffusion-weighted MRI (DWI) was able to reflect tumor biological features of PCNSL [20]. And other studies demonstrated that there were significant correlation between the MRI-based textures and tumor pathological process or tumor microenvironment [19, 21]. However, the consensus has not been reached due to the inadequate evidence, bringing the urgency of more researches in the future to investigate the relationship between specific texture features and tumor characteristics.

There are some limitations in our study. First and foremost, this is a retrospective study with variable time of follow-up periods. A prospective study is required to confirm the reliability of results. Secondly, the population involved in is relatively small even with a large database. The reason is that some patients came from remote areas and were hard to clinically follow in the long-term. Third, some of our results did not accord with previous studies, like the results of univariate analysis which showed that age was negatively significantly related to the patient survival in this study. Even with a similar result from another study, explanation seems not convincing [16]. Fourth, the prognostic value of TA was only applied for the texture features extracted from histo- and GLCM matrixes. The selection bias of 10 parameters used for this study is inevitable. Finally, there are several other pathological subtypes of PCNSL, but only DLBCL were enrolled in this study. The value of texture analysis in other pathological lymphoma subtypes should be verified in future research.

## Figures and Tables

**Table 1:** Characteristics of patients diagnosed with PCNSL.

Characteristics	Numbers	*p* value
Age	58.45 (range: 32∼75)	0.210
<60 years old	36 (69.2%)	
>60 years old	16 (30.8%)	
Gender		0.372
Male	29 (55.8%)	
Female	23 (44.2%)	
Surgery		0.684
Stereotactic biopsy (STB)	35 (57.7%)	
Subtotal resections (STRs)	17 (13.5%)	
Chemotherapy		
High-dose MTX-based chemotherapy regimen	49 (94.2%)	
R-CHOP+MTX chemotherapy regimen	3 (5.8%)	
Radiotherapy		0.032
Yes	11 (21.2%)	
No	41 (78.8%)	
Patient survival status		
Dead	18 (mean time: 22 months)	
Alive	34	

**Table 2:** Receiver operating characteristic curve analysis on MRI texture features.

MRI texture features	ROC threshold	*p* value	AUC	95% CI
Histo-Energy	>0.03	0.179	0.615	0.470 to 0.747
Histo-Entropy	≤1.54	0.120	0.634	0.489 to 0.763
Histo-Kurtosis	>4.07	0.093	0.642	0.497 to 0.770
Histo-Skewness	≤1.37	0.806	0.523	0.380 to 0.663
GLCM-Correlation	>0.36	0.155	0.614	0.469 to 0.746
GLCM-Contrast	**<121.17**	**0.016**	**0.681**	**0.538 to 0.804**
GLCM-Dissimilarity	**<7.81**	**0.015**	**0.681**	**0.538 to 0.804**
GLCM-Entropy	≤2.86	0.163	0.614	0.469 to 0.746
GLCM-Energy	>0.0018	0.087	0.636	0.491 to 0.765
GLCM-Homogeneity	**≥0.29**	**0.039**	**0.663**	**0.519 to 0.788**

ROC: receiver operating characteristic; AUC: area under curve; CI: confidence interval; GLCM: grey-level co-occurrence matrix.

**Table 3:** Multivariate Cox proportional hazard regression analysis on MRI texture parameters.

MRI texture features	HR	*p* value	95% CI
GLCM-Homogeneity	3.075	0.021	1.188∼7.957

HR: hazard ratio; CI: confidence interval; GLCM: grey-level co-occurrence matrix.

## Data Availability

The data used to support the findings of this study are available from the corresponding author upon request.

## Supplementary Materials

Supplementary Materials Supplement Table 1: Mann–Whitney *U* test on texture features extracted by two neurosurgeons.
