# Supplementary material for: Contrast-Enhanced MRI Texture Parameters as Potential Prognostic Factors for Primary Central Nervous System Lymphoma Patients Receiving High-Dose Methotrexate-Based Chemotherapy
Source: Contrast Media Mol Imaging. 2019 Nov 12;2019:5481491. doi: 10.1155/2019/5481491 (PMC6875177; doi:10.1155/2019/5481491)
Supplement: Supplementary Materials — Supplement Table 1: Mann–Whitney U test on texture features extracted by two neurosurgeons. [file 5481491.f1.docx]

Supplement Table 1: Mann-Whitney U test on texture features extracted by two neurosurgeons

| *MRI texture features* | *p value* |
| --- | --- |
| Histo-Energy | 0.956 |
| Histo-Entropy | 0.627 |
| Histo-Kurtosis | 0.862 |
| Histo-Skewness | 0.993 |
| GLCM-Correlation | 0.876 |
| GLCM-Contrast | 0.993 |
| GLCM-Dissimilarity | 0.660 |
| GLCM-Entropy | 0.848 |
| GLCM-Energy | 0.763 |
| GLCM-Homogeneity | 0.415 |

GLCM-: Grey-level co-occurrence matrix
